# Ensifentrine in COPD patients taking long-acting bronchodilators: A pooled post-hoc analysis of the ENHANCE-1/2 studies

**DOI:** 10.1177/14799731251314874

**Published:** 2025-01-24

**Authors:** Mark Dransfield, Nathaniel Marchetti, Ravi Kalhan, Daniel Reyner, Amy L Dixon, Tara Rheault, Kathleen Ann Rickard, Antonio Anzueto

**Affiliations:** 1 Medicine/Pulmonary, Allergy and Critical Care, 9968 University of Alabama at Birmingham and the Birmingham VA Medical Center, Birmingham, AL, USA; 2 Thoracic Medicine and Surgery, 12314 Lewis Katz School of Medicine at Temple University, Philadelphia, PA, USA; 3 Division of Pulmonary and Critical Care Medicine, Department of Preventive Medicine, Feinberg School of Medicine, 12244 Northwestern University, Chicago, IL, USA; 4 670719 Verona Pharma plc, Raleigh, NC, USA; 5 South Texas Veterans Health Care System, 11024 University of Texas Health, San Antonio, TX, USA

**Keywords:** Chronic obstructive pulmonary disease, ensifentrine, dual PDE3 and PDE4 inhibitor, maintenance therapy

## Abstract

**Background:**

The efficacy and safety of ensifentrine, a novel PDE3/PDE4 inhibitor, were previously evaluated in the ENHANCE-1 (NCT04535986) and ENHANCE-2 (NCT04542057) trials. Here, we present a pooled post-hoc subgroup analysis of patients according to background chronic obstructive pulmonary disease (COPD) maintenance medication regimens.

**Objective:**

This analysis aimed to explore the efficacy and safety of ensifentrine in patients receiving long-acting muscarinic antagonists (LAMA) or long-acting beta-agonists with inhaled corticosteroids (LABA + ICS).

**Methods:**

Eligible patients had moderate to severe COPD, were aged 40–80 years, and were symptomatic at randomization. Patients were randomized 5:3, receiving twice-daily ensifentrine 3 mg or placebo via standard jet nebulizer over 24 weeks.

**Results:**

The pooled post-hoc analysis included 485 LAMA patients and 272 LABA + ICS patients. Ensifentrine showed lung function improvement over placebo at week 12, including average FEV_1_ AUC_0–12 h_ in the LAMA (placebo-corrected least squares mean change from baseline [LSMC], 92 mL; 95% CI, 54, 131; *p* < 0.001) and LABA + ICS subgroups (LSMC, 74 mL; 95% CI, 27, 121; *p* = 0.002). Ensifentrine reduced the rate and risk of exacerbations in both LAMA (48% and 50%, respectively) and LABA + ICS (51% and 56%, respectively) subgroups. Ensifentrine-treated patients reported improvement in symptoms and quality of life over 24 weeks. The safety profile of ensifentrine in each subgroup was similar to the profile in the pooled modified intention-to-treat population.

**Conclusions:**

Nebulized ensifentrine offers a novel non-steroidal anti-inflammatory and bronchodilator treatment added to existing LAMA or LABA + ICS treatment options in patients with moderate to severe, symptomatic COPD.

## Introduction

Chronic obstructive pulmonary disease (COPD) is a leading cause of morbidity and mortality, and the prevalence is expected to increase globally.^1,2^ COPD is characterized by progressive airflow obstruction leading to daily symptoms that impact the quality of life as well as acute exacerbations, which can contribute to a decline in lung function over time.^3–5^ These negative outcomes emphasize the importance of interventions that decrease COPD symptoms and exacerbation burden.

Current maintenance regimens for patients with COPD include long-acting bronchodilators (long-acting muscarinic antagonists [LAMA] or long-acting beta-agonists [LABA]), which can be used together and be combined with inhaled corticosteroids (ICS). Despite multiple variations of approved therapies and combinations of bronchodilator and ICS therapies, patients continue to suffer from symptoms and exacerbations that impair their quality of life. Novel therapeutics with different yet complementary mechanisms of action present opportunities to impact exacerbations and symptoms. For example, for Medicare Advantage patients (*N* = 789) taking LAMA/LABA or LABA/ICS from 2017 to 2018, 44% of the patients had at least 1 moderate/severe exacerbation in the past year.^6^ Moreover, survey data from the US, Europe, Japan, and China reveal that 64% of patients taking dual or triple therapy for at least 6 months reported a significant burden of symptoms (COPD Assessment Test >20).^7^ Even with the addition of adjunctive non-bronchodilator–based therapy in certain COPD patient subgroups (i.e., azithromycin, roflumilast, and dupilumab), there is still a need to reduce symptoms and exacerbations, as all of these agents possess drawbacks associated with lack of efficacy in a broad COPD population, tolerability, cost, and the need for self-injection.^8–10^

Ensifentrine is a novel, first-in-class, selective, dual inhibitor of PDE3 and PDE4 that has demonstrated robust non-steroidal anti-inflammatory activity and bronchodilator effects.^11,12^ Dual inhibition of PDE3 and PDE4 has been shown to provide enhanced benefit in inducing relaxation of airway smooth muscle and suppression of the inflammatory response in cell cultures compared with inhibitors of PDE3 or PDE4 alone.^13–15^ In the phase 3 ENHANCE-1 and ENHANCE-2 trials of twice-daily ensifentrine 3 mg versus placebo in patients with moderate to severe COPD, either on no background maintenance therapy or taking LABA or LAMA therapy with or without ICS, ensifentrine significantly improved lung function and symptoms, and reduced the rate and risk of moderate and severe COPD exacerbations.^16^

It is important to assess the effect of ensifentrine, given its novel mechanism of action, when added on to existing classes of background medication. Here, we present a pooled post-hoc analysis from the ENHANCE-1 and ENHANCE-2 trials evaluating the efficacy of ensifentrine in subgroups of patients according to background COPD maintenance medication regimen.

## Methods

The ENHANCE-1 (EudraCT Number: 2020-002086-34; NCT04535986) and ENHANCE-2 (EudraCT Number: 2020-002069-32; NCT04542057) trials were phase 3, multicenter, randomized, double-blind, parallel-group, placebo-controlled studies in patients with symptomatic, moderate to severe COPD. The trial designs have been described in depth previously.^16^ Briefly, patients were randomized 5:3 to receive twice-daily ensifentrine (3 mg or placebo over 24 weeks via standard jet nebulizer [PARI, Starnberg, Germany]). A subset of patients in the ENHANCE-1 trial were randomized in a 3:1 ratio to receive treatment over 48 weeks. Randomization was stratified by treatment duration (ENHANCE-1 only; 24 or 48 weeks), stable background maintenance with LAMA or LABA (yes or no), and smoking status (current or former).

### Patients

The eligibility criteria for the ENHANCE-1 and ENHANCE-2 trials have been described previously.^16^ Briefly, eligible patients had moderate to severe COPD (FEV_1_/FVC <0.7, 30–70% predicted normal FEV_1_), were 40–80 years old, and had significant dyspnea at randomization (Modified Medical Research Council Dyspnea Scale score ≥2). Exclusion criteria included a history of asthma, life-threatening COPD, hospitalization for COPD, pneumonia, COVID-19 within 12 weeks of screening, and a record of a COPD exacerbation requiring oral or intravenous steroids within 3 months of screening. Patients taking stable maintenance regimens for at least 2 months prior to screening continued their established maintenance regimens throughout the trial (LAMA or LABA, with or without ICS). Initiation or cessation of COPD maintenance therapy after randomization was not permitted unless medically necessary. Other prohibited medications/therapies included steroid therapies, antibiotics, PDE4 inhibitors, LAMA/LABA combination medications, and oral β_2_-agonists. A complete list of prohibited therapies is provided in the online data supplement (Table S1).

### Procedures

The primary endpoints of the ENHANCE-1 and ENHANCE-2 trials were the effects of ensifentrine on lung function compared with placebo over a 12-h dosing interval (average FEV_1_ AUC_0–12 h_). Patients received an eDiary at screening for recording daily rescue medication use, Evaluating Respiratory Symptoms (E-RS) scores, and treatment adherence. The St. George’s Respiratory Questionnaire (SGRQ) and Transition Dyspnea Index (TDI) assessments were administered during clinic visits. Moderate COPD exacerbations were defined as worsening of COPD symptoms (≥2 major symptoms or 1 major and 1 minor symptom) for ≥2 days, requiring a minimum of 3 days of therapy with oral or systemic corticosteroids and/or antibiotics. Severe COPD exacerbations were defined as worsening symptoms that required inpatient hospitalization.

### Statistics

The individual ENHANCE trials were not powered for analyses of ensifentrine versus placebo within background medication subgroups for any endpoint. Thus, a pooled analysis was conducted to increase statistical power across FEV_1_, symptoms, quality-of-life, and exacerbation endpoints. Data were pooled from the ENHANCE-1 and ENHANCE-2 studies over 24 weeks to create pooled modified intention-to-treat and pooled safety populations. Both populations included all randomized patients who received at least 1 dose of the double-blind study medication.

A post-hoc pooled analysis was conducted to assess the effect of ensifentrine versus placebo on lung function, moderate/severe exacerbations, and patient-reported outcomes (E-RS total score, SGRQ total score, and TDI score at weeks 6, 12, and 24) in patients who were taking LAMA or LABA + ICS as maintenance therapy in addition to the assigned study treatment. Continuous endpoints were compared using an analysis of covariance model adjusting for the study, treatment, region, background medication strata, and smoking status as factors, with the baseline as the covariate. Data are presented as the least squares mean change from baseline. Missing data were imputed using multiple imputation techniques based on treatment, background medication strata, smoking strata, region, baseline, and when applicable, other post-dose time points for the endpoint, and based on the Missing-at-Random data assumption. The rate for moderate or severe exacerbations was compared between ensifentrine and placebo in each subgroup using a negative binomial model adjusting for the study, treatment, region, background medication strata, and smoking status, with the log study time (years) as an offset. Time to first exacerbation was analyzed using the Cox proportional hazards model, which included terms for the study, region, background medication strata, and smoking status. Summaries of the incidence of adverse events over 24 weeks provided the frequency and percentages of patients who experienced at least 1 event. Analyses were performed using SAS version 9.4 (SAS Institute, Cary, North Carolina).

### Trial oversight

The ENHANCE-1 and ENHANCE-2 trials were approved at each institution by independent ethics committees or review boards. The trials were performed in accordance with the Declaration of Helsinki and Good Clinical Practice. All patients provided written informed consent. Two trial sites in ENHANCE-1 and one in ENHANCE-2 were excluded from all analyses prior to database lock and unblinding due to significant non-compliance with good clinical practice.

## Results

### Patients

In total, 975 patients in the ensifentrine group and 574 patients in the placebo group were included in the pooled analysis (Figure S1). In the LAMA maintenance therapy subgroup (*n* = 485), 319 patients were randomized to ensifentrine and 166 patients were randomized to placebo. In the LABA + ICS subgroup (*n* = 272), 159 patients were randomized to ensifentrine and 113 patients were randomized to placebo (Table 1). Patients taking triple therapy (LAMA + LABA + ICS) or dual LAMA + LABA therapy were excluded from ENHANCE-1 and ENHANCE-2.

**Table 1.** Demographics and baseline disease characteristics.

Characteristic, *n* (% mITT)	mITT		LAMA		LABA + ICS	
	Ensifentrine *n* = 975	Placebo *n* = 574	Ensifentrine *n* = 319 (32.7)	Placebo *n* = 166 (28.9)	Ensifentrine *n* = 159 (16.3)	Placebo *n* = 113 (19.7)
Demographics, *n* (%)						
≥65 years of age	532 (54.6)	317 (55.2)	179 (56.1)	101 (60.8)	88 (55.3)	77 (68.1)
Female	457 (46.9)	269 (46.9)	138 (43.3)	79 (47.6)	82 (51.6)	51 (45.1)
Male	518 (53.1)	305 (53.1)	181 (56.7)	87 (52.4)	77 (48.4)	62 (54.9)
Region						
North America	374 (38.4)	238 (41.5)	67 (21.0)	40 (24.1)	65 (40.9)	53 (46.9)
European	589 (60.4)	325 (56.6)	243 (76.2)	121 (72.9)	92 (57.9)	56 (49.6)
Baseline disease characteristics						
Moderate COPD	561 (57.5)	307 (53.5)	180 (56.4)	99 (59.6)	85 (53.5)	48 (42.5)
Severe COPD	414 (42.5)	267 (46.5)	137 (42.9)	67 (40.4)	74 (46.5)	65 (57.5)
Exacerbation in 15 months prior to screening	220 (22.6)	136 (23.7)	98 (30.7)	50 (30.1)	38 (23.9)	32 (28.3)
Current smoker	544 (55.8)	323 (56.3)	163 (51.1)	80 (48.2)	75 (47.2)	53 (46.9)
Baseline assessment scores, mean (SD)						
E-RS total score	13.7 (6.7)	13.3 (6.1)	14.0 (7.0)	13.2 (6.1)	12.7 (6.6)	13.5 (5.8)
SGRQ total score	49.4 (17.9)	49.1 (16.9)	49.5 (17.5)	50.0 (16.9)	49.0 (18.7)	47.7 (17.8)
BDI score	5.9 (1.2)	5.9 (1.2)	6.1 (1.1)	6.0 (0.9)	5.6 (1.3)	5.8 (1.5)

BDI: baseline dyspnea index; COPD: chronic obstructive pulmonary disease; E-RS: evaluating respiratory symptoms; ICS: inhaled corticosteroid; LABA: long-acting beta-agonist; LAMA: long-acting muscarinic antagonist; mITT: modified intent-to-treat; SD: standard deviation; SGRQ: St. George’s respiratory questionnaire.

In the LAMA subgroup, 42.9% of patients treated with ensifentrine and 40.4% of patients treated with placebo had severe (GOLD 3) COPD. Similar proportions reported an exacerbation within 15 months of screening (ensifentrine-treated patients, 30.7%; placebo-treated patients, 30.1%) and were current smokers (ensifentrine-treated patients, 51.1%; placebo-treated patients, 48.2%). In the LABA + ICS subgroup, 46.5% of patients treated with ensifentrine and 57.5% of patients treated with placebo had severe COPD. Rates of exacerbations within 15 months of screening (ensifentrine-treated patients, 23.9%; placebo-treated patients, 28.3%) and the proportion of current smokers (ensifentrine-treated patients, 47.2%; placebo-treated patients, 46.9%) were similar between patients treated with ensifentrine and placebo.

### Efficacy

**Figure 1.**
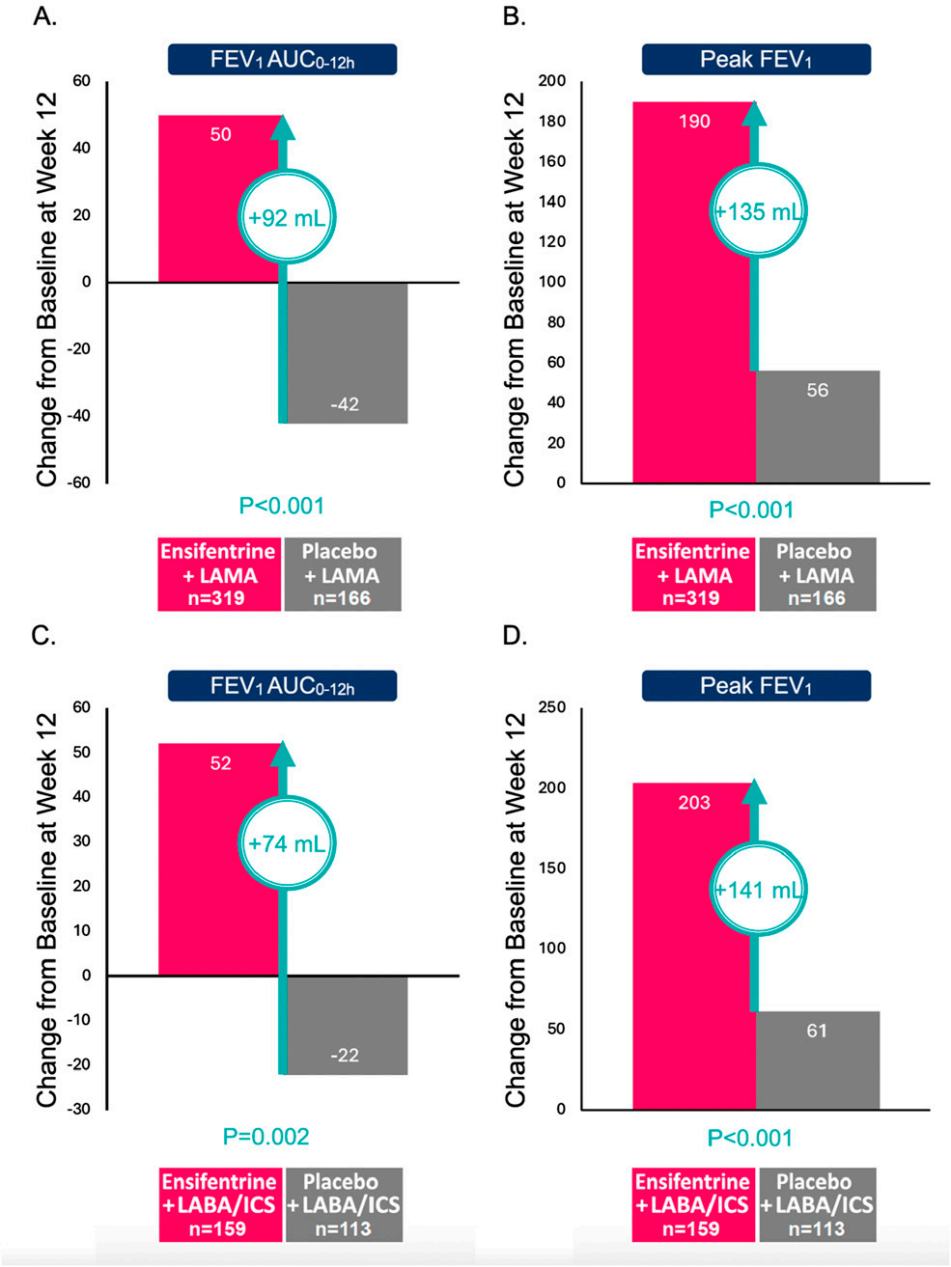
Difference in least squares mean change from baseline estimates (ensifentrine vs placebo) at week 12 in lung function. Note: Differences shown versus placebo have been rounded. AUC_0–12 h_: area under the effect versus time curve from 0 to 12 h; FEV_1_: forced expiratory volume in 1 s; ICS: inhaled corticosteroid; LABA: long-acting beta-agonist; LAMA: long-acting muscarinic antagonist; LS: least squares; SE: standard error.

In this pooled post-hoc analysis, the improvement in lung function as measured by between-group differences in the least squares mean change from baseline (LSMC) for FEV_1_ AUC_0–12 h_, peak FEV_1_, and morning trough FEV_1_ provided by ensifentrine was consistent across both background medication subgroups. Specifically, among patients on LAMA background therapy, ensifentrine showed improvement over placebo at week 12 in the average FEV_1_ AUC_0–12 h_ (LSMC, 92 mL; 95% CI, 54, 131; *p* < 0.001; Figure 1(a)), peak FEV_1_ (LSMC, 135 mL; 95% CI, 91, 179; *p* < 0.001; Figure 1(b)), and morning trough FEV_1_ (LSMC, 44 mL; 95% CI, 2, 86; *p* = 0.039; data not shown). Similarly, among patients on LABA + ICS background therapy, ensifentrine showed improvements over placebo for the average FEV_1_ AUC_0–12 h_ (LSMC, 74 mL; 95% CI, 27, 121; *p* = 0.002; Figure 1(c)), peak FEV_1_ (LSMC, 141 mL; 95% CI, 89, 194; *p* < 0.001; Figure 1(d)), and morning trough FEV_1_ (LSMC, 59 mL; 95% CI, 12, 106; *p* = 0.015; data not shown).

**Figure 2.**
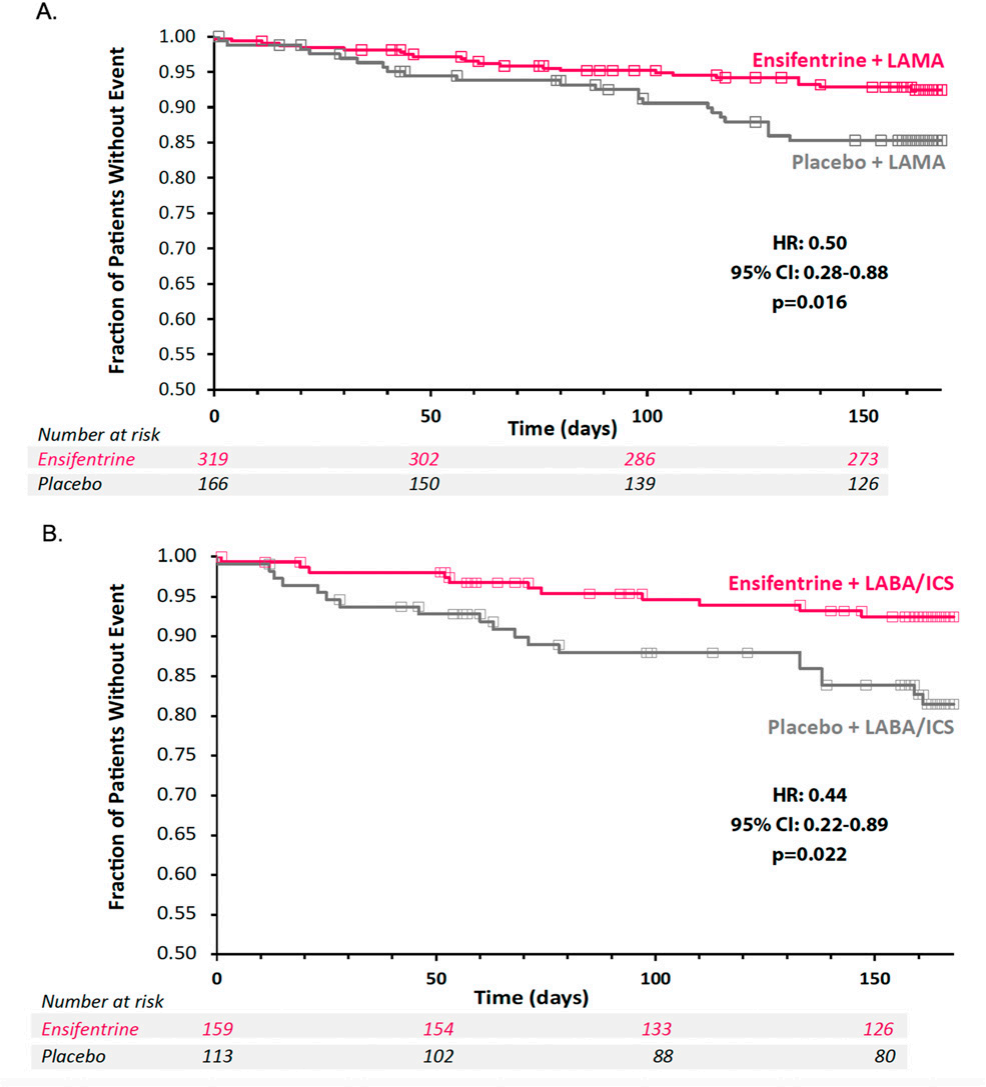
Rate and risk of moderate*/severe^†^ exacerbations over 24 weeks by medication subgroup. *Moderate exacerbations were defined as worsening of COPD symptoms (≥2 major symptoms or 1 major and 1 minor symptom) for ≥2 days, requiring a minimum of 3 days of therapy with oral or systemic corticosteroids and/or antibiotics. ^†^Severe COPD exacerbations were defined as the worsening of specified COPD symptoms that required inpatient hospitalization. COPD: chronic obstructive pulmonary disease; HR: hazard ratio; ICS: inhaled corticosteroid; LABA: long-acting beta-agonist; LAMA: long-acting muscarinic antagonist.

Moderate/severe exacerbation rate and risk (measured by time to first event) were assessed over 24 weeks in both background medication subgroups. In the LAMA subgroup, ensifentrine-treated patients showed a 48% reduction in the moderate/severe exacerbation rate (rate ratio [RR], 0.52; 95% CI, 0.30, 0.92; *p* = 0.023) and a 50% reduction in risk, as measured by time to first event (hazard ratio [HR], 0.50; 95% CI, 0.28, 0.88; *p* = 0.016) compared with placebo (Figure 2(a)). In the LABA + ICS subgroup, ensifentrine-treated patients showed a 51% reduction in the moderate/severe exacerbation rate (RR, 0.49; 95% CI, 0.24, 0.99; *p* = 0.046) and a 56% reduction in risk (HR, 0.44; 95% CI, 0.22, 0.89; *p* = 0.022) compared with placebo (Figure 2(b)).

### Patient-reported outcomes

Among patients in both background medication subgroups, COPD symptoms improved in ensifentrine-treated patients versus placebo throughout the 24-week study period as assessed by multiple independent patient-reported measures (Table 2). Ensifentrine-treated patients who were on LAMA background therapy reported an improvement over placebo in dyspnea, as measured by TDI scores as early as week 6 that were sustained through week 24 (least squares mean difference [LSMD], 1.16; 95% CI, 0.59, 1.73; *p* < 0.001). Ensifentrine-treated patients who were taking LABA + ICS reported improvements over placebo in TDI scores at week 24 (LSMD, 0.77; 95% CI, 0.05, 1.49; *p* = 0.037), with numerical improvements at earlier time points. Further, improvements in symptoms as measured by LSMC in the E-RS total score exceeding the minimum clinically important difference of −2 units were observed for ensifentrine-treated patients in the LAMA subgroup at weeks 6 through 24, which were statistically greater than placebo at weeks 6 and 12, and numerically greater at week 24 (week 24 LSMD, −0.93; 95% CI, −1.90, 0.03; *p* = 0.058). Numerical improvements over placebo, as measured by LSMC in E-RS total scores, were noted in patients who received ensifentrine in the LABA + ICS subgroup at weeks 6 and 12. Quality of life, as measured by St. George’s Respiratory Questionnaire, was numerically improved over placebo at all time points in those who received ensifentrine, whether they were on LAMA (week 24 LSMD, −2.41; 95% CI, −5.04, 0.22; *p* = 0.072) or LABA + ICS (week 24 LSMD, −1.65; 95% CI, −5.05, 1.76; *p* = 0.344) background medication (Table 2).

**Table 2.** Change from baseline in patient-reported outcomes at weeks 6, 12, and 24 by medication subgroup.

	LAMA		LABA + ICS	
	Ensifentrine	Placebo	Ensifentrine	Placebo
TDI^a^				
Patients, *n*	313	164	155	109
Week 6				
LS mean (SE)	1.62 (0.25)	0.73 (0.28)	1.09 (0.38)	0.75 (0.39)
LS mean difference (95% CI)	0.90 (0.44, 1.35)		0.34 (−0.28, 0.95)	
*p* value	<0.001		0.281	
Week 12				
LS mean (SE)	1.68 (0.27)	0.67 (0.30)	0.93 (0.43)	0.39 (0.44)
LS mean difference (95% CI)	1.02 (0.53, 1.50)		0.55 (−0.13, 1.22)	
*p* value	<0.001		0.113	
Week 24				
LS mean (SE)	2.38 (0.30)	1.22 (0.35)	1.39 (0.47)	0.63 (0.50)
LS mean difference (95% CI)	1.16 (0.59, 1.73)		0.77 (0.05, 1.49)	
*p* value	<0.001		0.037	
E-RS^b^				
Patients, *n*	317	166	158	113
Week 6				
LS mean (SE)	−2.43 (0.43)	−1.57 (0.48)	−1.45 (0.69)	−0.33 (0.70)
LS mean difference (95% CI)	−0.87 (−1.66, −0.08)		−1.12 (−2.21, −0.04)	
*p* value	0.032		0.042	
Week 12				
LS mean (SE)	−2.60 (0.47)	−1.64 (0.53)	−1.42 (0.69)	−0.57 (0.70)
LS mean difference (95% CI)	−0.96 (−1.83, −0.09)		−0.86 (−1.93, 0.22)	
*p* value	0.031		0.119	
Week 24				
LS mean (SE)	−2.68 (0.52)	−1.75 (0.58)	−1.18 (0.80)	−1.18 (0.81)
LS mean difference (95% CI)	−0.93 (−1.90, 0.03)		0.001 (−1.22, 1.22)	
*p* value	0.058		0.999	
SGRQ^c^				
Patients, *n*	316	166	156	111
Week 6				
LS mean (SE)	−6.39 (1.26)	−5.00 (1.42)	−7.73 (1.84)	−4.85 (1.88)
LS mean difference (95% CI)	−1.40 (−3.76, 0.97)		−2.88 (−5.81, 0.05)	
*p* value	0.248		0.054	
Week 12				
LS mean (SE)	−7.59 (1.27)	−5.42 (1.43)	−3.38 (1.89)	−1.96 (1.93)
LS mean difference (95% CI)	−2.17 (−4.54, 0.20)		−1.42 (−4.38, 1.53)	
*p* value	0.073		0.345	
Week 24				
LS mean (SE)	−8.05 (1.40)	−5.63 (1.58)	−2.83 (2.24)	−1.19 (2.31)
LS mean difference (95% CI)	−2.41 (−5.04, 0.22)		−1.65 (−5.05, 1.76)	
*p* value	0.072		0.344	

E-RS: evaluating respiratory symptoms; ICS: inhaled corticosteroid; LABA: long-acting beta-agonists; LAMA: long-acting muscarinic antagonist; LS: least squares; MCID: minimum clinically important difference; SE: standard error; SGRQ: St. George’s respiratory questionnaire; TDI: transition dyspnea index.

^a^MCID = 1.

^b^MCID = −2.

^c^MCID = −4.

### Safety

Overall, the safety profile for ensifentrine was similar to that of placebo across the subgroups of background maintenance regimens (Table 3). The rates of treatment-emergent adverse events (TEAEs) over 24 weeks were similar between the patients treated with ensifentrine and placebo across the pooled modified intention-to-treat safety population (36.8% vs 35.9%), the LAMA subgroup (33.9 % vs 31.9%), and the LABA + ICS subgroup (41.5% vs 45.1%). The most prevalent TEAEs over 24 weeks in the LAMA subgroup were COVID-19 infection (ensifentrine, 2.8% vs placebo, 1.2%), nasopharyngitis (2.5% vs 2.4%), and back pain (2.8% vs 0.6%); the most prevalent TEAEs over 24 weeks in the LABA + ICS subgroup were COVID-19 infection (7.5% vs 8.0%), hypertension (3.8% vs 1.8%), and headache (3.1% vs 3.5%; Table 4).

**Table 3.** Summary of treatment-emergent adverse events by medication subgroup.

Events, *n* (%)	Pooled mITT		LAMA		LABA + ICS	
	Ensifentrine *n* = 975	Placebo *n* = 574	Ensifentrine *n* = 319	Placebo *n* = 166	Ensifentrine *n* = 159	Placebo *n* = 113
Treatment-emergent AE	359 (36.8)	206 (35.9)	108 (33.9)	53 (31.9)	66 (41.5)	51 (45.1)
Treatment-related AE	44 (4.5)	23 (4.0)	9 (2.8)	10 (6.0)	8 (5.0)	3 (2.7)
Serious treatment-emergent AE	60 (6.2)	36 (6.3)	17 (5.3)	10 (6.0)	12 (7.5)	10 (8.8)
Serious treatment-related AE	1 (0.1)	0	0	0	1 (0.6)	0
Severe treatment-emergent AE	49 (5.0)	27 (4.7)	11 (3.4)	4 (2.4)	10 (6.3)	8 (7.1)
Treatment-emergent AEs leading to drug discontinuation	74 (7.6)	47 (8.2)	17 (5.3)	10 (6.0)	16 (10.1)	18 (15.9)
Treatment-emergent AEs leading to study discontinuation	54 (5.5)	30 (5.2)	11 (3.4)	5 (3.0)	14 (8.8)	14 (12.4)
Treatment-emergent AEs leading to death	6 (0.6)	5 (0.9)	2 (0.6)	1 (0.6)	1 (0.6)	2 (1.8)

AE: adverse event; ICS: inhaled corticosteroid; LABA: long-acting beta-agonists; LAMA: long-acting muscarinic antagonist; mITT: modified intent-to-treat.

**Table 4.** Most frequent (≥2% in any group) TEAEs.

Events, *n* (%)	Pooled mITT		LAMA		LABA + ICS	
	Ensifentrine *n* = 975	Placebo *n* = 574	Ensifentrine *n* = 319	Placebo *n* = 166	Ensifentrine *n* = 159	Placebo *n* = 113
COVID-19	34 (3.5)	21 (3.7)	9 (2.8)	2 (1.2)	12 (7.5)	9 (8.0)
Headache	26 (2.7)	19 (3.3)	3 (0.9)	6 (3.6)	5 (3.1)	4 (3.5)
Nasopharyngitis	22 (2.3)	19 (3.3)	8 (2.5)	4 (2.4)	2 (1.3)	8 (7.1)
Back pain	18 (1.8)	6 (1.0)	9 (2.8)	1 (0.6)	2 (1.3)	3 (2.7)
COPD worsening	18 (1.8)	11 (1.9)	3 (0.9)	3 (1.8)	2 (1.3)	4 (3.5)
Hypertension	17 (1.7)	5 (0.9)	2 (0.6)	0	6 (3.8)	2 (1.8)
Upper respiratory tract infection	10 (1.0)	7 (1.2)	2 (0.6)	1 (0.6)	2 (1.3)	3 (2.7)
Pneumonia	8 (0.8)	6 (1.0)	2 (0.6)	1 (0.6)	2 (1.3)	3 (2.7)
Dyspnea	6 (0.6)	8 (1.4)	1 (0.3)	4 (2.4)	1 (0.6)	3 (2.7)
Dizziness	4 (0.4)	6 (1.0)	1 (0.3)	0	2 (1.3)	3 (2.7)

COPD: chronic obstructive pulmonary disease; ICS: inhaled corticosteroid; LABA: long-acting beta-agonists; LAMA: long-acting muscarinic antagonist; mITT: modified intent-to-treat; TEAE: treatment-emergent adverse event.

## Discussion

PDE3 and PDE4 are promising targets for COPD, as both are expressed in airway and inflammatory cells, and regulate the second messenger molecules cyclic adenosine monophosphate and cyclic guanosine monophosphate. Dual inhibition of PDE3 and PDE4 reduces bronchial tone in airway smooth muscle,^17–19^ mediates inflammatory cell activation/migration and stimulates the cystic fibrosis transmembrane conductance regulator.^20–24^ Ensifentrine, a novel dual inhibitor of PDE3 and PDE4, added on to background LAMA or LABA + ICS maintenance therapy demonstrated consistent improvements across lung function, symptom, and quality of life endpoints compared with placebo. Notably, ensifentrine also reduced moderate and severe exacerbation rate and risk compared with patients treated with LAMA alone (with placebo) or LABA + ICS (with placebo). This post-hoc subgroup analysis supports that ensifentrine, a dual PDE3/PDE4 inhibitor, provides additional benefit to standard classes of COPD therapy consistent with its novel mechanism of action. Clinically meaningful bronchodilation, symptom improvement, and exacerbation reduction were shown with ensifentrine added on to LAMA or LABA + ICS therapy, supporting complementary bronchodilatory and anti-inflammatory effects to these therapies.

Ensifentrine demonstrated an improvement over placebo across both background maintenance therapy subgroups for average FEV_1_ AUC_0–12 h_, peak FEV_1_, and morning trough FEV_1_. The magnitude of effect observed from ensifentrine on exacerbation reduction when added on to LAMA or LABA + ICS therapy was substantial and offers a novel, non-steroidal mechanism for meaningful additive effects in patients with moderate and severe COPD treated with LAMA or LABA + ICS therapy. It is well accepted that reductions in the rate and risk of exacerbations can have clinical implications beyond the immediate prevention of the escalation of symptoms, as exacerbation history is a strong predictor of future exacerbations and is associated with increased rates of lung function decline.^4,25^ Notably, previous studies that have shown a reduction in acute exacerbations of COPD included populations that were enriched for patients who experience frequent exacerbations,^26^ whereas this study was conducted in a population that was representative of a broad COPD population.

Ensifentrine-treated patients who were taking LAMA or LABA + ICS reported improvements over placebo in TDI at week 24 that exceeded the minimum clinically important difference, indicating a profound impact on dyspnea. Additionally, ensifentrine-treated patients in the LAMA subgroup reported clinically meaningful improvements in symptoms as assessed by E-RS and TDI scores at all time points. Notably, directional changes were consistent across all symptom and quality of life measures at all weeks in both LAMA and LABA + ICS subgroups, with the only exception of the E-RS total score in the LABA + ICS subgroup at week 24, in which increased effects in the placebo group resulted in similar improvements to the ensifentrine group. Overall, these findings highlight the complementary mechanism of ensifentrine, which shows clinically important effects in addition to commonly used classes of maintenance therapies.

The safety profile of ensifentrine over 24 weeks in each subgroup examined was similar to that observed in the pooled modified intention-to-treat population. The overall rate of TEAEs was low and similar across each of the examined treatment groups in both background medication subgroups. Further, the most common TEAEs were generally consistent between the ensifentrine and placebo groups in both background medication subgroups. No safety concerns of note for ensifentrine were identified among patients receiving concomitant LAMA or LABA + ICS.

This post-hoc analysis has several strengths, including the pooled population resulting in a larger analysis population for each background medication subgroup and the inclusion of multiple, independent patient-reported outcomes assessed over 24 weeks. A limitation of this study is that, due to the post-hoc nature of the analysis, conclusions on statistical significance for endpoints described herein cannot be made. Another limitation is the exclusion of patients receiving triple therapy (LAMA + LABA + ICS) and patients on dual LAMA + LABA therapy, as the ENHANCE trials were designed to inform on the efficacy and safety of ensifentrine with its novel mechanism of action added on to each class of inhaled maintenance therapy (e.g., LAMA, LABA, and ICS). Furthermore, LAMA monotherapy and LABA + ICS remain commonly utilized maintenance treatments for COPD in current practice (27, 28), with LAMA monotherapy continuing to be recommended by GOLD.^4^

In conclusion, nebulized ensifentrine offers a novel non-steroidal anti-inflammatory and bronchodilator treatment that provides a complementary and new mechanism that can be added to existing LAMA or LABA + ICS treatment options in patients with moderate to severe, symptomatic COPD. Ensifentrine has been shown to provide substantial reductions in rate and risk of exacerbations and improvements in lung function and symptoms of COPD, particularly dyspnea, along with improvements in quality of life when added to patients who were already receiving standard therapy with LAMA or LABA + ICS.

## Supplemental Material

Supplemental Material - Ensifentrine in COPD patients taking long-acting bronchodilators: A pooled post-hoc analysis of the ENHANCE-1/2 studies Supplemental Material for Ensifentrine in COPD patients taking long-acting bronchodilators: A pooled post-hoc analysis of the ENHANCE-1/2 studies by Mark Dransfield, Nathaniel Marchetti, Ravi Kalhan, Daniel Reyner, Amy L Dixon, Tara Rheault, Kathleen Ann Rickard and Antonio Anzueto in Chronic Respiratory Disease
