# Supplementary material for: Ensifentrine in COPD patients taking long-acting bronchodilators: A pooled post-hoc analysis of the ENHANCE-1/2 studies
Source: Chron Respir Dis. 2025 Jan 24;22:14799731251314874. doi: 10.1177/14799731251314874 (PMC11760128; doi:10.1177/14799731251314874)
Supplement: Supplemental Material - Ensifentrine in COPD patients taking long-acting bronchodilators: A pooled post-hoc analysis of the ENHANCE-1/2 studies [file sj-pdf-1-crd-10.1177_14799731251314874.pdf]

Ensifentrine in COPD Patients Taking Long-Acting Bronchodilators: A Pooled Post-hoc Analysis of the EHHANCE-1/2 Studies

Supplemental Material

Table S1. Prohibited Medications and Therapies in the ENHANCE Trials

Medication	Time Interval
Oral, Systemic, or Parenteral Steroid Therapies	3 months prior to Screening Visit and prohibited during the study.[a]
Antibiotics for lower respiratory tract infection	6 weeks prior to Screening Visit and prohibited during the study.[a] Chronic use of antibiotics was not allowed 6 weeks prior to or during the study.
Inhaled Corticosteroids (ICS) (e.g., ICS monotherapy and subjects in the no maintenance therapy stratum)<br>High dose ICS (e.g., >1000 mcg of fluticasone propionate or equivalent)	4 weeks prior to Screening Visit and prohibited during the study.
Oral leukotriene inhibitors (i.e., montelukast, zafirlukast, zileuton)	48 hours prior to Screening Visit and prohibited during the study.
Theophylline and PDE4 inhibitor (e.g., roflumilast, apremilast, crisaborole)	48 hours prior to Screening Visit and prohibited during the study.
Terbutaline	1-day prior to Screening Visit and prohibited

	during the study.
Ipratropium (including combinations with albuterol/salbutamol)	6 hours prior to Screening Visit and prohibited during the study.
LAMA/LABA combination products	4 weeks prior to Screening Visit and prohibited during the study.
Nebulized LAMA or LABA or ICS<br>For subjects in the no maintenance therapy stratum:<br>For subjects in the LAMA or LABA stratum:	 4 weeks prior to Screening Visit and prohibited during the study.<br>1-week prior to Screening Visit and prohibited during the study (e.g., subject switched from nebulized to dry powder or metered dose therapy)
LAMA Excluded ONLY for subjects in the no maintenance therapy stratum	4 weeks prior to Screening Visit and prohibited during the study.[b]
LABA Excluded ONLY for subjects in the no maintenance therapy stratum	4 weeks prior to Screening Visit and prohibited during the study.[b]
Oral β_2-agonists	1-week prior to Screening Visit and prohibited during the study.

[a]Except for the treatment of COPD exacerbations during the study. Localized corticosteroid injections (e.g., intra-articular and epidural), intranasal and topical corticosteroids were permitted.

[b]Washout of patients on stable background maintenance LAMA or LABA monotherapy for the sole purpose of study eligibility was not recommended.

COPD, chronic obstructive pulmonary disease; ICS, inhaled corticosteroids; LABA, long-acting β_2 agonist; LAMA, long-acting muscarinic antagonist; PDE4, phosphodiesterase 4.

Figure S1. CONSORT Flow Chart of the Pooled Population in ENHANCE-1 and ENHANCE-2

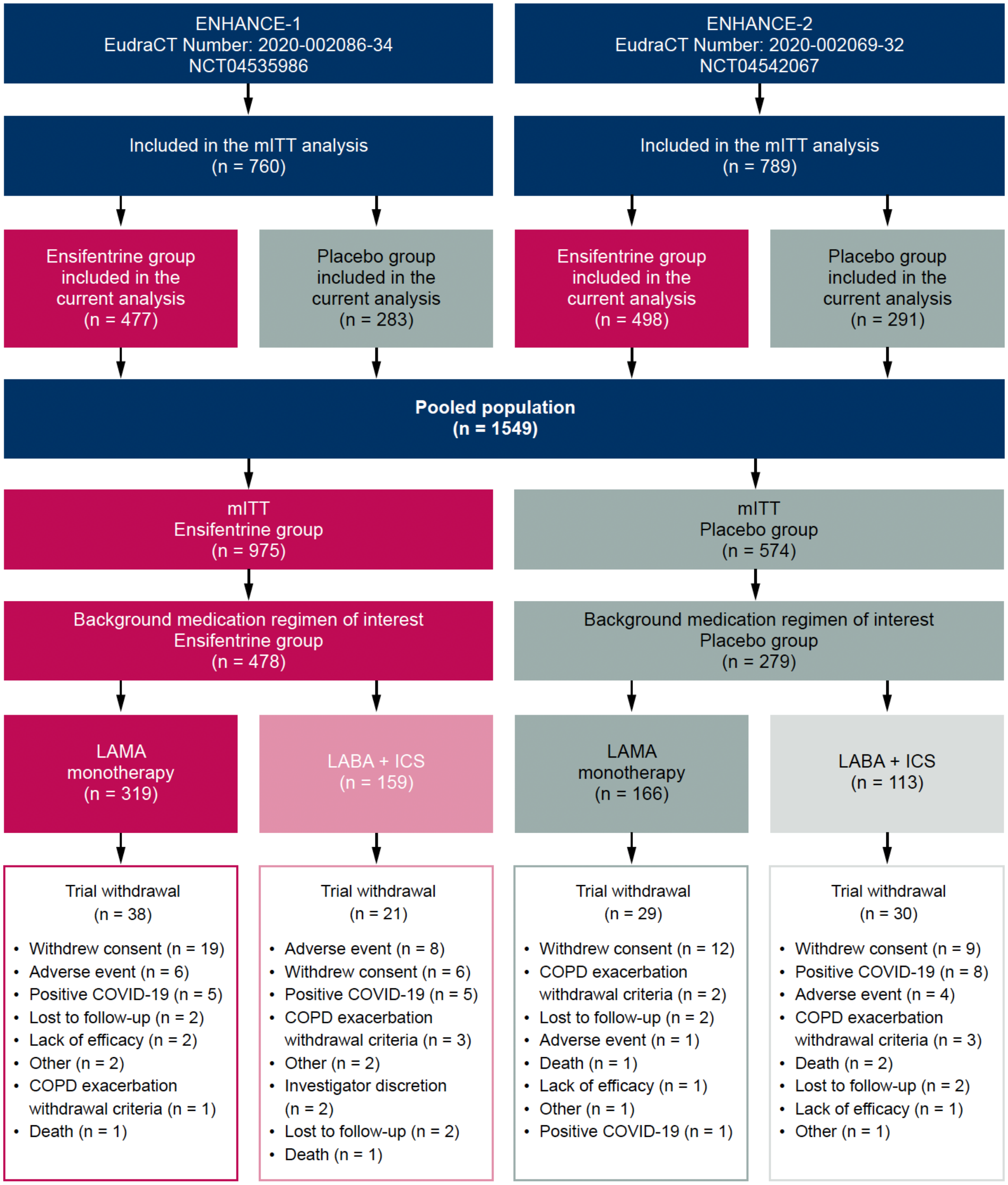

LABA, long-acting beta-agonist; LAMA, long-acting muscarinic antagonist; mITT, modified intent-to-treat.
